# Combining nanoscale manipulation with macroscale relocation of single quantum dots

**DOI:** 10.3762/bjnano.3.36

**Published:** 2012-04-10

**Authors:** Francesca Paola Quacquarelli, Richard A J Woolley, Martin Humphry, Jasbiner Chauhan, Philip J Moriarty, Ashley Cadby

**Affiliations:** 1 The Department of Physics and Astronomy, The Hicks Building, The University of Sheffield, Hounsfield Rd, Sheffield, South Yorkshire, S3 7RH, UK; 2 School of Physics and Astronomy, University of Nottingham, Nottingham, NG7 2RD UK; 3 Phase Focus Limited, The Kroto Innovation Centre, The University of Sheffield, North Campus, Broad Lane, Sheffield, S3 7HQ, UK

**Keywords:** automation, nanoscale manipulation, nanotechnology, quantum dots, single molecule spectroscopy

## Abstract

We have controllably positioned, with nanometre precision, single CdSe quantum dots referenced to a registration template such that the location of a given nanoparticle on a macroscopic (≈1 cm^2^) sample surface can be repeatedly revisited. The atomically flat sapphire substrate we use is particularly suited to optical measurements of the isolated quantum dots, enabling combined manipulation–spectroscopy experiments on a single particle. Automated nanoparticle manipulation and imaging routines have been developed so as to facilitate the rapid assembly of specific nanoparticle arrangements.

## Introduction

Techniques such as scanning probe microscopy and transmission electron microscopy have been used extensively to provide crucial high-resolution structural and morphological information on nanoscale systems. Measurement of the optical properties of a nanostructured material or nanoscale device with a resolution comparable to the length scale of the system of interest, however, continues to present a challenge. A number of techniques have been developed to push the resolution of optical microscopy and spectroscopy to the single-molecule/particle limit. These include scanning near-field optical microscopy (SNOM) [1–3] and techniques based on adaptations of single-molecule spectroscopy [4], such as fluorescence imaging with one-nanometer accuracy (FIONA) [5], stochastic optical reconstruction microscopy (STORM) [6]. These techniques require the fluorophore under observation to be isolated by distances larger than the diffraction limit of the microscope. The study of single fluorophores separated by distances larger than the diffraction limit has proven to be a valuable tool in understanding the optical properties of a broad range of nanostructured systems, including conjugated polymers [7], biomolecules [8–9], and quantum dots [10]. Nonetheless, these techniques fundamentally rely on a statistical distribution of molecules and are therefore not optimal for the study of specific isolated nanostructures at well-defined locations on a surface. Recent attempts at the positioning of quantum dots (QDs) based on electro-osmotic flow control (EOFC) [11–12] have resulted in a positioning precision of 130 nm when particle diffusion is suppressed. In a challenging recent experiment, atomic force microscopy (AFM) was used to manipulate a single gold nanoparticle (≈35 nm) to approach a single quantum dot [13]. The gold nanoparticle was shown to dramatically quench the optical lifetime of the QD and to completely suppress its blinking.

## Experimental

**Figure 1:**
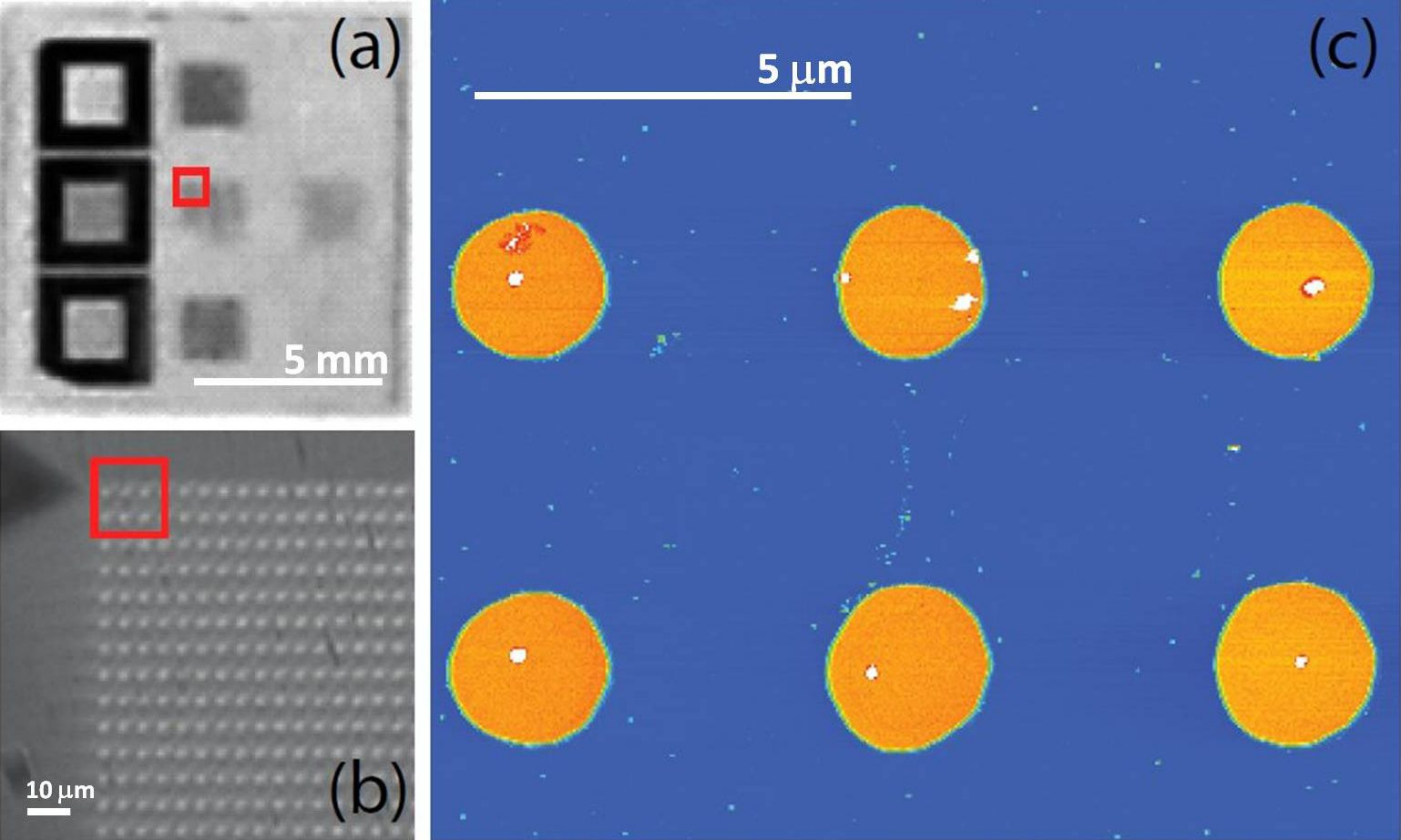
(a) An optical image of nine different reference grids on a 10 × 10 mm^2^ sapphire substrate; (b) Optical zoom showing the grid structure comprising gold pillars 2 μm in diameter, 75 nm tall, with a centre-to-centre distance of 5 μm; (c) AFM image of a number of the grid cells. Quantum dots (QDs) are visible as small dots in the image.

In this work, we position a single fluorophore, a CdSe QD, with nanometre precision on a macroscopic registration template, using automated atomic force microscopy (AFM) techniques. Our approach differs from previous work [13] in that we have manipulated, and subsequently re-located and spectroscopically identified, single nanoparticles of less than 5 nm diameter on an atomically flat substrate. Sapphire was selected as a suitable substrate as it is transparent across most of the visible spectrum [14] and comprises wide, atomically flat terraces (200 nm across) separated by steps only 0.22 nm in height. Moreover, as sapphire is an excellent electrical insulator it shows minimal quenching of optically excited states [13,15]. A simple optical lithography patterning procedure allows us to (re-)locate a given, previously positioned, QD in any optical system. To manipulate and characterise a single quantum dot we required the ability to repeatedly address an area of only a few square nanometres on a 10 × 10 mm^2^ substrate. In order to realise this relocation capability, the sapphire substrate was patterned by using a grid of gold pillars as shown in Figure 1a.

The center-to-center distance of the pillars is 5 μm and the pillar height 75 nm. Four pillars form a single cell, which can be repeatedly addressed in both an optical microscope and by AFM by simply assigning each cell an X, Y coordinate. An AFM height image for cells A1–B1, using our reference notation detailed below, is shown in Figure 1c This enabled the re-location of single nanoparticles so that they could be analysed using a number of different techniques including optical microscopy, single-molecule microscopy, and atomic force microscopy. Importantly, the samples discussed here were transferred between two laboratories separated by ≈50 km, but it was possible to study the same nanoparticle in both labs. The quantum dots used in this work were CdSe/ZnS core/shell hydrophobic nanocrystals (PlasmaChem [16]), coated with hydrophobic organic molecules (TOPO and HDA [16]) and with a maximum emission wavelength of 610 nm. To prepare a sample suitable for manipulation experiments, the nanocrystals were dispersed in HPLC-grade toluene and the concentration varied until a QD number density of ≈10 QD per square micron on the patterned sapphire substrate was achieved. For AFM imaging and manipulation we used an Asylum MFP-3D atomic force microscope in tapping mode (imaging) or contact mode (manipulation) with AC240TS Olympus AFM cantilevers. Several cells were imaged over a large scan, typically 20–40 μm in size. From these initial scans a cell that had a small number of suitable quantum dots (and showed little adsorbed contamination) was selected for the manipulation region.

**Figure 2:**
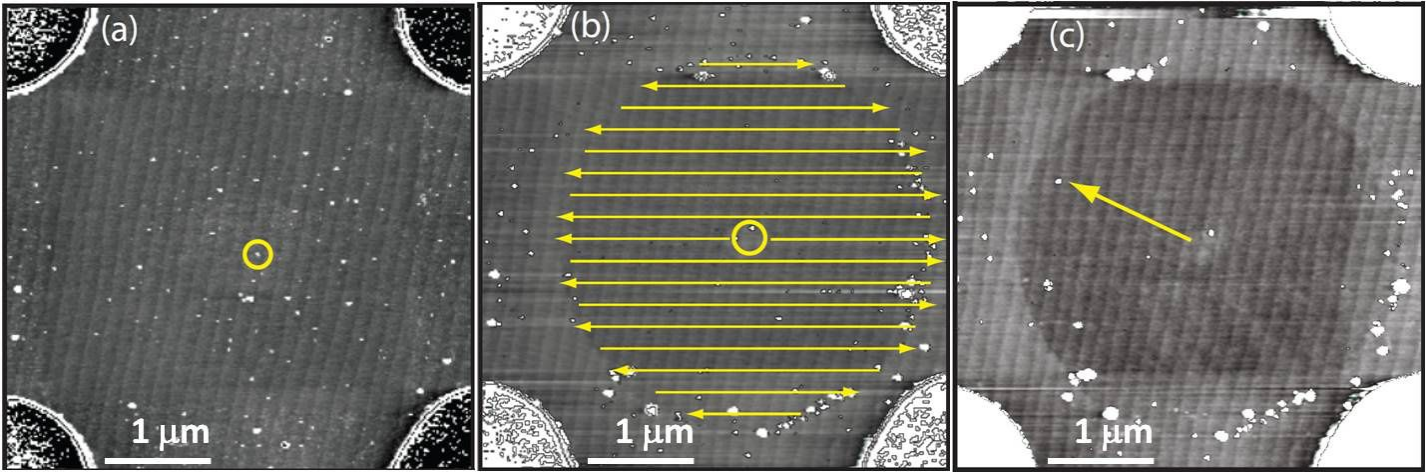
Isolation and manipulation of a single QD. (a) A typical cell after QDs have been spin cast on to the surface. The QD selected for study is highlighted with a yellow circle; (b) The yellow lines show the path that the AFM tip takes in contact mode, clearing a circle around the selected QD. (c) An area around the QD is cleared leaving only two QDs in the centre of the cell. The final QD is removed by nudging the QD with the AFM tip in contact mode with a high tip–sample interaction force. The approximately parallel lines seen in each of the images are atomic step edges on the sapphire substrate.

For a specific manipulation experiment, an individual QD having a line profile (height/lateral extent) appropriate for a single CdSe nanoparticle (as ascertained statistically from measurements of a large number of QDs) was selected, ensuring that it was at least one micron from the pillars and preferably close to the centre of the cell, as shown in Figure 2. The remaining unwanted QDs were moved away from the selected QD by using the AFM as follows: A suitable bitmap image was used as a mask to define the area of the substrate that was to be subjected to the *clearing* process, as shown in Figure 2b [17]. (A circular or square bitmap with an aperture centred on the selected QD was typically used). The AFM then performed a contact mode sweep in the area covered by the bitmap. The bitmap defined the areas in which the contact mode setpoint was high, i.e., an increased tip–sample interaction force is present. Figure 2b shows the regions of high contact force and the direction of travel of the AFM probe. Areas for which the bitmap was transparent corresponded to regions in which a low tip–sample interaction was required (e.g., close to the QD of interest). The use of manipulation masks of this type enabled a large area of the sample to be swept free of QDs quickly, leaving only the QD of interest (Figure 2c). Finally, and if required, the remaining QD can be nudged into place by using the AFM tip or a QD near to the QD of interest can be moved.

## Results and Discussion

**Figure 3:**
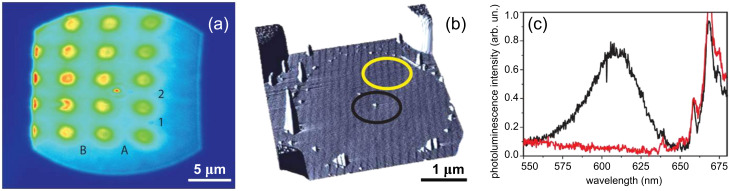
(a) A white-light image of several cells. The laser spot can be seen in cell A2; (b) An AFM image of cell B1. The black circle shows a single isolated QD, while the yellow circle indicates an area that has been cleared of QDs; (c) Photoluminescence spectrum from the areas of the cell highlighted in (b) after excitation with the 442 nm line of a He:Cd laser. The 610 nm QD emission can be clearly seen in the black spectrum. The emission above 650 nm is due to chromium contamination in the sapphire substrate.

Optical measurements of the isolated QD were taken on a custom single-molecule spectrometer as described in the work of Khalil et al. [18]. A white-light image of the sample was used to identify the cell of interest, Figure 3a. (Without the gold registration markers it would of course be practically impossible to identify the manipulated QDs on any microscope other than the AFM used to perform the manipulation). Figure 3a shows the superposition of a white-light image and a laser-spot image taken at the same time, in which the laser spot can be seen in cell A2. The image shown in Figure 3a is of the same grid cell as that in Figure 1a. An AFM image of cell B1 is shown in Figure 3b in which all of the QDs except one have been removed from the cell, leaving a single dot in the centre.

To investigate the optical properties of single QDs after manipulation, the sample was excited with the 442 nm line of a He:Cd laser, this produced a 1 μm laser spot on the surface, as shown in Figure 3a. The sample was translated until the laser spot overlapped with the know position of the manipulated QD. The slit width of the monochromator was reduced to allow only light from the laser spot to enter, and the QD photoluminescence was collected. The sample was then translated by 1 μm to a section of the sample that had been cleaned of QDs, and a second spectrum was taken from that area. The two corresponding spectra are shown in Figure 3c. Both spectra contain several sharp features between 650 nm and 750 nm arising from photoluminescence (PL) caused by the sapphire substrate and are likely due to chromium ions [19]. The PL spectrum taken on the manipulated QD is centred at 608 nm, and luminescence at this wavelength is completely absent elsewhere in the cell. The full-width at half-maximum (FWHM) of the QD spectra is 108 meV, which corresponds well to the previously studied emission from single colloidal quantum dots, for which PL blinking was recorded to optically identify the emission as being that from a single QD [20–21]. Due to limitations in the collection optics it was not possible to see blinking in our sample.

The PL contamination, caused by chromium defects in the sapphire, limits the use of this substrate to QDs with peak emission wavelengths below 650 nm or above 750 nm. We have also manipulated and imaged other QDs with peak emission wavelengths at 440 nm, 550 nm and 780 nm on sapphire. It was seen that the manipulation of the QD does not affect the QD emission: QDs that have been moved over several microns in the clearing process still emit at the expected central wavelength and are highly luminescent.

**Figure 4:**
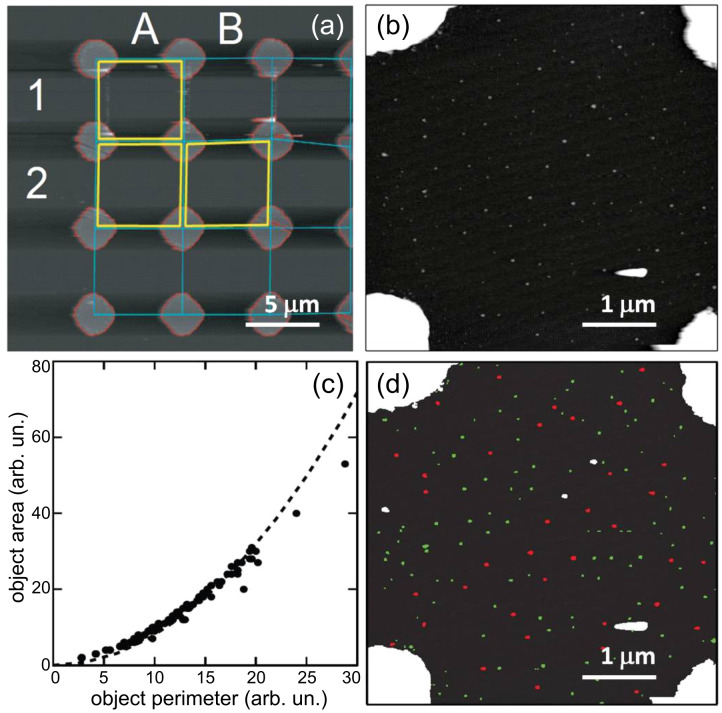
(a) The automation algorithm identifies the experimental cells from the macroscopic reference grid, (b) then zooms in. Each object over a given height threshold is classified according to its dimensions (c) (the dotted line indicates the area:perimeter ratio of a circle). The result of this classification is given in (d): larger noncircular features are classified as contaminations or QD clumps (white), whereas single quantum dots are given a red (larger) or green (smaller) colouration (indicating their position in the QD size distribution).

The manipulation of each quantum dot requires several hours of intensive AFM work by a highly skilled operator, and thus this is hardly a cost-effective, scalable process. To address this issue, we have taken steps to automate the process of identifying the registration template and experimental cells; identifying the nano-particles suitable for manipulation; and, finally, identifying the correct parameters to perform the manipulation itself. Figure 4a shows how the automation software locates the coordinates of the experimental cells, then zooms in and identifies the cell contents (Figure 4c and Figure 4d).

We employ a simple object-classification algorithm, which identifies particles on the substrate surface based on their topographic dimensions. As QDs have very specific heights and ratios of surface-area to volume, it is possible to set strict parameters on what can be considered a suitable single QD and what can be classed as a clump of QDs or contamination. This process can be performed iteratively, which allows for a re-evaluation of the sample after each manipulation and means that the system can identify manipulations that have resulted in a break up of a cluster or a manipulation that has not successfully manipulated a single QD. Our manipulation routines are also quite uncomplicated, using the AFM in contact mode rather than lift mode, allowing us to maintain feedback as the nanoparticles are pushed. Importantly, we can ascertain the minimum amount of surface–tip contact force required for the manipulation to take place; reducing tip wear and image degradation. Tip state also plays an important role in the manipulation process, and the automatic characterisation and optimization of the AFM tip apex would be beneficial, as has been shown for STM imaging [22].

By using this method it is possible to greatly increase the number of manipulations that can be completed, and it allows for the possibility of performing manipulations to create an individual and distinctive structure in each cell without the need for an operator. As the colour of a QD is size-dependent, the manipulation algorithm could be used with dual-colour samples in order to build up structures in which energy transfer is utilized.

## Conclusion

We have shown that it is possible to isolate and manipulate individual CdSe quantum dots on a sapphire surface and subsequently relocate the same QD within a macroscopic (centimetres squared) area to measure its optical properties. We have also taken initial steps pto scale the process by computer automation with possible applications in the fabrication of nanoscale devices.
